# Effect of TEAS combined with general anesthesia on early postoperative cognitive function in elderly patients undergoing single-port thoracoscopic lobectomy

**DOI:** 10.12669/pjms.38.8.6927

**Published:** 2022

**Authors:** Xi Chen, Haoliang Cai, Jun Guo

**Affiliations:** 1 Xi Chen, Department of Anesthesiology, Shuguang Hospital Affiliated to, Shanghai University of Traditional Chinese Medicine, Shanghai, 201203, China; 2 Haoliang Cai, Department of Anesthesiology, Shuguang Hospital Affiliated to, Shanghai University of Traditional Chinese Medicine, Shanghai, 201203, China; 3 Jun Guo, Department of Anesthesiology, Shuguang Hospital Affiliated to, Shanghai University of Traditional Chinese Medicine, Shanghai, 201203, China

**Keywords:** NSCLC, TEAS, Cognitive function, Single port thoracoscopic lobectomy

## Abstract

**Objective:**

To investigate the effect of transcutaneous electrical acupoint stimulation (TEAS) on early cognitive function in elderly patients after single port thoracoscopic lobectomy.

**Methods:**

One hundred and nine patients who underwent single whole thoracoscopic lobectomy in Shuguang Hospital Affiliated to Shanghai University of Traditional Chinese Medicine from January 2020 to October 2021 were included in this single-center, retrospective observational study. According to the treatment records, before anesthesia, 56 patients received TEAS (TEAS-group), and 53 patients applied electrodes at the same acupoint without electrical stimulation (control-group). Preoperative and postoperative cognitive function (Mini mental state examination, MMSE score), serum neuron specific enolase (NSE), S100β protein and p-tau protein levels and postoperative complications were compared between the two groups.

**Results:**

After operation, the MMSE of the TEAS-group was significantly better than that of the Control-group, with the MMSE of the TEAS-group returned to the preoperative level 72 hours after operation. Serum NSE, S100β and p-tau concentrations 24 hours and 72 hours after operation in the TEAS-group returned to their preoperative level and were significantly lower than those in the control-group. Hospitalization time of the TEAS-group was significantly shorter and hospitalization expenses were significantly lower comparing to the control-group.

**Conclusion:**

TEAS treatment could promote improved early postoperative cognitive function of elderly patients undergoing single port thoracoscopic lobectomy and could accelerate the recovery.

## INTRODUCTION

With the increasing life expectancy and the increased incidence of lung cancer, more elderly patients are requiring treatment for non-small cell lung cancer (NSCLC). While surgery is still the primary treatment of lung cancer,^1^ the development of endoscopic technology gradually changed a surgical approach from thoracotomy to thoracoscopic surgery.^2^ Early postoperative cognitive dysfunction is one of the most prominent and concerning perioperative complications that affects 47% of elderly patients treated for non-cardiac surgery.^3^,^4^ The pathogenesis of early postoperative cognitive dysfunction has not been determined yet, but may be related to the cholinergic response, the stress response and the inflammatory response.^4^

To date, there is no evidence showing the effectiveness of drug prevention strategies in reducing the occurrence of early postoperative cognitive dysfunction in elderly patients. Transcutaneous electrical acupoint stimulation (TEAS) is mainly a combination of electrical stimulation therapy and the traditional meridian theory of traditional Chinese medicine, which uses acupoint conduction to stimulate and subsequently improve the functional status of patients.^5^ Clinical studies have found that TEAS therapy can effectively improve immunological function during the perioperative period in patients with NSCLC^6^, and improve the neurological function and local microcirculation in patients following a stroke.^7^

TEAS therapy can relieve clinical symptoms, improve hand dysfunction, and seems to further improve overall treatment of stroke patients.^7^,^8^ TEAS can also regulate the function of human organs and the activity of sympathetic nerve. Using electrical stimulation, TEAS can effectively regulate the sympathetic nerve through promoting nervous system balance and improve vascular permeability, ultimately enhancing the metabolism of the body.^9^ In recent years, TEAS has shown a good effect in the clinical treatment of a variety of diseases.^10^ This protocol has a unique role in organ and brain protection, and anti-inflammatory response. Therefore, TEAS may reduce the occurrence of early postoperative cognitive dysfunction in elderly patients.

At present, the combination of TEAS with anesthesia is widely used in clinical practice as studies have found that TEAS could reduce postoperative analgesic requirement in patients undergoing surgery and expedite patients’ recovery.^7^,^11^,^12^ Unlike traditional acupuncture and electro acupuncture, TEAS is a non-invasive acupoint electrical stimulation. There are many advantages of TEAS such as a low infection rate, controllable parameters and a straightforward operation.^13^ Additionally, TEAS can inhibit the over activation of the immune system 14 and reduce the ischemia and hypoxia injury of the nervous system during and post-operation.^8^ The aim of this study was to investigate the effect of TEAS on early postoperative cognitive function in elderly (>60 years old) patients undergoing single port thoracoscopic lobectomy.

## METHODS

In this single-center, retrospective observational study, the records of 109 patients (69 males and 40 females) who underwent single whole thoracoscopic lobectomy in Shuguang Hospital Affiliated to Shanghai University of Traditional Chinese Medicine from January 2021 to October 2021 were reviewed. Before anesthesia, 56 patients received TEAS and were set as TEAS-group, and 53 patients applied electrodes at the same acupoint without electrical stimulation and were set as control-group. The study was approved by the hospital ethics committee (No. 20220103) and informed consent was taken from all patients.

### Inclusion criteria:

- Age>60 years
- Single port thoracoscopic lobectomy was performed
- Body mass index (BMI): 20 kg/m^2^ < BMI ≤ 30 kg/m^2^
- Complete medical records

### Exclusion criteria:

- History of surgery within the past year
- Serious complications resulting from surgery, central nervous system diseases or severe mental health diseases before the operation
- History of drug allergy before operation
- Long term oral administration of psychotropic or opioid drugs before operation
- Allergic patients with intolerance to TEAS

TEAS was initiated 30min before anesthesia. Acupoint selection Taiyang xue and Fengchi xue (according to the national standard of the people’s Republic of China GB 12346-90).^11^ Operation Electrodes were placed at Taiyang and Fengchi xue and the electrodes were connected with a G6805-2 electro acupuncture instrument (produced by Shanghai Huayi Medical Instrument Co., Ltd.). The wave type was density wave, 2/100Hz, peak current 5mA. The induction of general anesthesia began 30 minutes after TEAS stimulation. Following tracheal intubation, the electrical stimulation intensity gradually increased to 7-7.5 mA peak current, and electrical stimulation was conducted until the end of operation.

### Anesthesia procedure:

Patients were monitored for noninvasive arterial blood pressure, peripheral capillary oxygen saturation (SpO2) and echocardiograms upon arrival at the operating room. Intravenous anesthesia induction was performed with propofol 1.5~2.5mg/kg, sufentanil 0.4μg/kg, and cisatracurium 0.2mg/kg. After successful induction of anesthesia, tracheal intubation was performed, and mechanical ventilation was started by connecting to the anesthesia machine. During the process, the oxygen flow rate, tidal volume and respiratory rate were 2 L/ minutes, 8–10 ml/kg and 10–14 times/ minutes, respectively. Anesthesia was maintained by target-controlled infusion (TCI) of remifentanil 1~4 ng/ml, propofol 1~4 μg/ml and continuous infusion of cisatracurium 0.06 mg/(kg·h) to keep the bispectral index (BIS) at 40~60 and the anesthesia nociception index(ANI) at 50~70. Sufentanil 0.1 μg/kg was given at the 30mins before the end of the operation for analgesia transition and 0.375% ropivacaine 20ml was given for intercostal nerve block upon skin closure. After that, the patient was returned to the postanesthesia care unit and was extubated when the indication for extubation was met.

Patient-controlled intravenous analgesia (PCIA) was performed with sufentanil for all patients for two days. The analgesic drug formulation was Sufentanil 200μg + flurbiprofen 300mg, with normal saline to make up to 200 ml (priming volume 0ml, single dose 3ml, background infusion 2ml/h and lockout interval 15 mins). The numeric rating scale (NRS) score were recorded and NRS score at rest and deep breathing was maintained at less than four.

In the control-group, the electrodes were placed 2-cm beside Taiyang and Fengchi xue, 30min before anesthesia, without electrical stimulation. The anesthesia process was the same as that of the TEAS-group.

The patients laid on their side, with their chest properly raised and slightly tilted forward. The affected upper limb was abducted and fixed. After confirming that the contralateral lung ventilation was good, routine disinfection and towel application were carried out. The surgical incision was between the anterior axillary line and the middle axillary line. The surgical incision of the upper lobe and the middle lobe was in the fourth intercostal space of the affected side. The incision of the lower lobe was in the fifth intercostal space. The length of the incision was about 3cm. After cutting the skin layer by layer, the intercostal muscle was opened, followed by opening of the pleura and entering the chest cavity. A soft incision protective sleeve was placed at the incision. Under the assistance of laparoscopy, the pulmonary ligament was released, the pulmonary artery, pulmonary vein and bronchus were separated in turn, lobectomy and lymphadenectomy were performed, and the thoracic closed drainage tube was placed at the incision.

NES, S100β and p-tau were measured 24 hours before the operation and again 24 hours and 72 hours following the operation. Test method: venous blood was collected (3ml) and centrifuged at 3000r/minutes with a 3cm centrifugation radius at 4°C for five minutes. The supernatant was stored at -20°C. Prior to measurement, the blood sample was thawed for 30min. The concentrations of NES, S100β and p-tau in plasma were determined by enzyme-linked immunosorbent assay using Shanghai Kehua ST-360 microplate reader. The relevant kits were purchased from Wuhan bode Bioengineering Co., Ltd.

Cognitive function was assessed by mini mental state examination (MMSE), 24 hours before the operation and 24- and 72 hours after the operation. The MMSE examination was conducted by trained nurses who are blinded to the information about grouping and acupoints. The MMSE included five orientation items: memory, attention and calculation, recall ability and language ability, with 30 excitement points. The higher the score, the better the cognitive function.

### Intraoperative and postoperative records:

operation time, intraoperative blood loss, hospitalization time, treatment cost, consumption of remifentanil, propofol and cisatracurium, extubation time, NRS score, PCIA press numbers and postoperative complications (pulmonary infection, postoperative bleeding, atelectasis, and postoperative hypoxemia) were recorded.

Statistical analysis was done using SPSS 25.0 software. The measurement data were expressed by mean±standard deviation. The difference between groups was assessed using a t-test and variance analysis, respectively, and the count data was tested by χ^2^ test. *P*<0.05 was considered statistically significant.

## RESULTS

There was no significant difference between groups in the age or ratio of male to female patients, pathological type of lung cancer or coexisting diseases (Table-I, *P*>0.05). The hospitalization time of the TEAS-group was significantly shorter than that of the Control-group, resulting in a significantly lower overall hospitalization cost (*P*<0.05). However, there was no significant difference in operation time and intraoperative blood loss between the two groups (Table-II, *P*>0.05).

**Table-I:** Baseline data of two groups.

Index		Control (n=53)	TEAS (n=56)	t/χ^2^	p
Age (years, mean ± SD)		71.86±7.24	70.82±7.19	0.756	0.451
Gender (n/%)	Male	31(58.49%)	38(67.86%)	1.028	0.311
	Female	22(41.51%)	18(32.14%)		
Pathological type (n/%)	Squamous cell carcinoma	25(47.17%)	21(37.50%)	1.591	0.451
	Adenocarcinoma	13(24.53%)	13(23.21%)		
	Large cell carcinoma	15(28.30%)	22(39.29%)		
Coexisting diseases (n/%)	Other pulmonary diseases	8(15.09%)	11 (19.64%)	0.391	0.532
	Hypertension	19(35.85%)	25(44.64%)	0.875	0.350
	Diabetes	16(30.19%)	18(32.14%)	0.048	0.826
	Heart disease	20(37.73%)	24(42.86%)	0.297	0.586

TEAS: transcutaneous electrical acupoint stimulation.

**Table-II:** Operation and hospital parameters compared between the control and TEAS-groups.

Index	Control (n=53)	TEAS (n=56)	t	p
Operation time (min)	182.37±20.81	183.84±18.81	-0.387	0.699
Intraoperative blood loss (ml)	76.37±5.84	74.65±5.48	1.582	0.117
Postoperative hospital stay (d)	4.9±0.64	4.4±0.98*	3.467	0.001
Treatment costs (10000 RMB)	7.56±0.77	7.05±0.56*	3.907	<0.001

Data is mean ± standard deviation. TEAS: transcutaneous electrical acupoint stimulation,

* p<0.05 vs control-group

Pre-operatively, there was no difference in MMSE scores between the two groups (Table-III, *P*>0.05). Post-operatively (24 hours) there was a decrease in MMSE scores in both groups, with a greater decrease observed in the Control-group (Table-III, *P*<0.001). Post-operatively (72 hours), the patients in the TEAS-group recovered their MMSE scores to pre-operative level (Table-III, *P*<0.001).

**Table-III:** Pre- and post-operative comparison of MMSE scores between the control and TEAS-groups.

Group	Pre-operative 24h	Post-operative 24h	Post-operative 72h
Control (n=53)	27.55±0.82	24.16±1.25#	26.46±1.03#
TEAS (n=56)	27.79±0.64	25.76±1.51 *#	27.47±0.91*
t	-1.480	-5.483	-4.251
p	0.142	<0.001	<0.001

Data is mean ± standard deviation. TEAS: transcutaneous electrical acupoint stimulation.

* p<0.05 vs Control-group.

# p<0.05 vs. Preoperative 24h group.

Pre-operatively, there was no significant difference in serum NSE, S100β and p-Tau concentrations between the two groups (Table-IV, *P*>0.05). The serum levels of NSE, S100β and p-Tau were increased 24 hours and 72 hours after operation in both groups, with significantly higher concentrations of all markers in the control-group (Table-IV, *P*<0.05). Post-operatively (72 hours), the patients in the TEAS-group recovered their serum levels of NSE, S100β and p-Tau to pre-operative concentrations (Table-IV, *P*<0.05).

**Table-IV:** Pre- and post-operative comparison of serum NSE, S100β and p-Tau levels between the control and TEAS-groups.

	Group	Pre-operative 24h	Post-operative 24h	Post-operative 72h
NSE (ug/L)	Control	7.27±0.86	10.84±1.18#	7.98±0.60#
	TEAS	7.51±0.96	9.38±0.76*#	7.51±0.78*
	t	-1.319	7.658	2.351
	p	0.190	<0.001	0.021
S100β (ug/L)	Control	1.03±0.13	1.46±0.18i#	1.18±0.15i#
	TEAS	1.02±0.11	1.39±0.13*#	1.08±0.11*
	t	0.159	2.402	3.932
	p	0.874	0.018	<0.001
p-Tau (ug/L)	Control	89.24±5.13	138.94±12.19i#	94.62±4.24i#
	TEAS	90.62±5.09	128.20±10.94*#	92.02±6.44*
	t	-1.409	4.841	2.471
	p	0.162	<0.001	0.015

Data are mean ± standard deviation. TEAS: transcutaneous electrical acupoint stimulation.

* p<0.05 vs Control-group.

# p<0.05 vs Pre-operative 24h group.

There was one case of pulmonary infection, two cases of post-operative hemorrhage, three cases of post-operative hypoxemia and three cases of atelectasis in the Control-group (Table-V). In TEAS-group, there were one case of pulmonary infection, two cases of post-operative hemorrhage, two cases of post-operative hypoxemia and one case of atelectasis (Table-V). There was no significant difference in the incidence of post-operative complications between the two groups (Table-V, *P*>0.05).

**Table-V:** Comparison of complications between the control and TEAS-groups.

Group	Pulmonary infection	Post-operative hemorrhage	Post-operative hypoxemia	Atelectasis	Incidence of complications
Control (n=53)	1 (1.89%)	2 (3.77%)	3 (5.66%)	3 (5.66%)	9 (16.98%)
TEAS (n=56)	1 (1.78%)	2 (3.57%)	2 (3.57%)	1 (1.78%)	6 (10.71%)
t/χ^2^					0.901
p					0.342

TEAS: transcutaneous electrical acupoint stimulation.

## DISCUSSION

The results of this study showed that the hospitalization time, treatment cost and serum NSE, S100β and p-Tau levels of the TEAS treatment group were significantly lower than those of the control-group, while the MMSE score was significantly higher than that of the control-group. TEAS treatment did not significantly increase post-operative complications, indicating that this method is suitable for elderly patients with single port thoracoscopic lobectomy, and can effectively promote the recovery of cognitive function in these patients.

In the study by Chen J et al.^15^, investigating sedative and postoperative analgesic effects of TEAS on lung cancer patients undergoing thoracoscopic pneumonectomy, 80 patients were randomly divided into TEAS group and Sham TEAS combined general anesthesia group. The results showed that patients in the TEAS group had significantly lower visual analogue scale (VAS) scores at six, 24, and 48 hours after surgery (*P*<0.01); lower BIS at 10, 20, and 30 minutes before induction (*P*<0.01); lower levels of postoperative sufentanil consumption; lower number of PCIA attempts and effective rates (*P*<0.01); lower incidences of nausea at zero, six, 24, and 48 hours; and lower incidence of vomiting at 24 hours after surgery (*P*<0.05). The postoperative Observer’s Assessment of Alertness/Sedation (OAAS) scores were similar between the groups.

It shows that TEAS is a feasible method of sedation and postoperative analgesia. Similarly, Xiong W et al.^7^ showed that TEAS, administered in combination with local anesthesia during carotid artery stenting, can inhibit transient increases in carotid artery stenting, reduce the incidence of postoperative. Randomized controlled trial by Yeh ML et al.^16^ showed that TAES is a noninvasive, simple, and convenient modality for post-hemorrhoidectomy-associated pain control and anxiety reduction. Although the indicators observed in this study are different from the above studies, our results show that TEAS treatment can promote the improvement of patients’ condition and accelerate their rehabilitation after surgery.

At present, MMSE is adapted from the Mini Mental State Examination. This test can comprehensively, quickly and accurately reflect patients’ mental state and determine the degree of cognitive impairment. Because of its high sensitivity and specificity, MMSE is suitable for the evaluation of post-operative cognitive status in elderly patients.^17^ Study have shown that the peak of post-operative cognitive dysfunction is 1-3 days after surgery.^18^ In our study, the scores of both groups of patients 24 hours before the operation were compared with the scores 24 and 72 hours after the operation.

There was no significant difference in MMSE scores between the two groups. However, MMSE scores of patients in both groups were significantly decreased 24 hours post-operation, with a greater decrease observed in the control-group. Patients in the TEAS-group showed a recovery of MMSE scores to pre-operative levels 72h post-operation. This suggests that TEAS can significantly improve the early cognitive function of elderly patients after a single port thoracoscopic lobectomy.

NSE is a cytoplasmic protein with enolase activity, which is synthesized and secreted by cerebral neurons.^19^ S100β protein is a neurotrophic protein secreted by cerebral glial cells.^20^ Both proteins play a major role in learning, memory and cognitive function and their blood levels increase when the permeability of brain nerve cells or the blood-brain barrier is damaged. Studies suggest that serum NSE and S100β protein indexes can be used as biochemical markers to judge and evaluate the degree and prognosis of brain injury.^21^ Our results showed that serum NSE and S100β protein levels in the TEAS-group were significantly lower than those in the control-group, suggesting that TEAS can improve post-operative brain injury in elderly patients.

Certain pathological conditions result in abnormal phosphorylation of tubulin associated unit (Tau) protein that plays a role in maintaining the stability of microtubules in axons.^22^ Some studies have shown that abnormal p-Tau levels indicate the formation of neurofibrillary tangles in the brain parenchyma.^23^ Many clinical studies have confirmed that the concentration of p-Tau protein in cerebrospinal fluid of patients with Alzheimer’s disease is significantly higher than that of healthy people.^22^

Other studies have shown that the improvement of cognitive function in patients with epilepsy may be related to the decrease of p-Tau protein level.^24^ Our results showed that the level of serum p-Tau protein in the TEAS-group was significantly lower than that in the control-group, suggesting that TEAS may improve post-operative cognitive function.

### Limitation of the study:

It is a single center with a small sample size, and TEAS was only performed before surgery. However, Sun K et al.^25^ shows that a combination of preoperative TEAS with intraoperative or postoperative TEAS for management of postoperative pain following laparoscopic surgery is more effective than preoperative TEAS alone. Also, the cognitive profile of patients coexisted with COPD and asthma, which could result in cognitive dysfunction^26^, were not considered. Therefore, future studies should take it into account.

## CONCLUSION

TEAS, combined with general anesthesia, can promote early post-operative cognitive function of elderly patients with single port thoracoscopic lobectomy, and can accelerate the post-operative recovery.

### Authors’ contributions:

**XC** conceived and designed the study. **HC** and **JG** collected the data and performed the analysis. **XC** was involved in the writing of the manuscript and is responsible for the integrity of the study. All authors have read and approved the final manuscript.
